# Exploratory factor analysis of post traumatic stress disorder checklist for DSM-5: investigating post traumatic stress disorder interconnected dynamics with depression and anxiety in the aftermath of multiple collective stressors

**DOI:** 10.1371/journal.pone.0323422

**Published:** 2025-05-08

**Authors:** Josleen Al Barathie, Elie G. Karam

**Affiliations:** 1 Institute for Development, Research, Advocacy, and Applied Care (IDRAAC), Beirut, Lebanon; 2 Department of Psychiatry and Clinical Psychology, Saint George University Beirut, Beirut, Lebanon; 3 Department of Psychiatry and Clinical Psychology, St George Hospital University Medical Center, Beirut, Lebanon; Central South University, CHINA

## Abstract

Post Traumatic Stress Disorder (PTSD), a psychological condition linked to traumatic events, sees continuous Diagnostic Statistical Manual (DSM) criteria adjustments. The PTSD Checklist (PCL-5) is a favored screening tool for its versatility. Exploratory and Confirmatory Factor Analysis (EFA/CFA) studies on PCL-5’s utility across languages and settings reveal varied models, with conflicting outcomes attributed to methodological differences. Prompted by lack of research on PCL-5 in populations exposed to multiple collective traumas, we aim to conduct an EFA among a Lebanese nationally representative sample. To our knowledge, this study represents the first attempt at PCL-5 construct validation in Arabic. Recruitment involved 1,000 Lebanese adults aged 18 +. EFA with Promax rotation, aimed to identify the minimal number of factors among PCL-5 items, supported by Kaiser-Meyer-Olkin (KMO) and Bartlett’s test of sphericity. Eigenvalues above 1 indicate appropriateness for EFA with two retained factors (trauma specific vs non-trauma specific), supported by the scree plot with loadings ranging from 0.532 to 0.775. Understanding PCL-5’s structural factors is crucial, as it directly impacts diagnostic algorithms in clinical and research settings and informs knowledge about PTSD’s comorbidities. This, in turn, affects disorder prevalence, etiology, and intervention formulation. Our paper offers suggestions to tackle PCL-5 performance amidst comorbidity, categorized into DSM and non-DSM related recommendations. DSM-related recommendations include grouping PTSD, depression, and anxiety into a single “distress” disorder or eliminating non-specific PTSD symptoms. Non-DSM-related recommendations propose a shift towards a transdiagnostic approach and advocating for nuanced clinical interviews over self-report questionnaires to address symptom complexity and potential double counting in individuals with significant emotional distress.

## Introduction

Post-Traumatic Stress Disorder (PTSD) is a potentially disabling psychological condition that distinguishes itself from the majority of psychological disorders by virtue of possessing a distinctly defined and specific etiology secondary to a traumatic event or a significant stressor [1].

PTSD was formally incorporated as a diagnosable condition within the Diagnostic and Statistical Manual of Mental Disorders (3rd edition) [2]. To meet the criteria for PTSD, an individual had to undergo an exceptionally severe and uncommon traumatic event in war zones. Nowadays, there is a broadened range that include non-combat experiences during periods of peace such as motor vehicle accidents, rape, intimate partner violence, natural disasters, and childhood abuse.

While filling a much-needed gap in both theory and practical application, DSM-III was influenced by political considerations and driven by theoretical perspectives [3,4]. In subsequent DSM editions (DSM-III-R, DSM-IV, and DSM-IV-TR), PTSD criteria underwent continuous debates, reconceptualization, and modifications [5–8].

Nowadays, DSM-5 PTSD endured structural adjustments [1]. Initially classified as an anxiety disorder, PTSD has now been moved beyond a narrow fear-based anxiety disorder and positioned within the Trauma- and Stressor-related Disorders category [9]. While controversial, this change was based on research [10–14]. Furthermore, the symptoms are now organized into four sets “*B,C,D and E*” instead of the previous three “*reexperiencing (Criterion B), avoidance/numbing (Criterion C), and hyperarousal (Criterion D)*” [1,6,15].

The DSM-5 introduced a new criterion D by integrating most of the DSM-IV criterion C symptoms, except for effortful avoidance, which has been reclassified as DSM-5 C1 and C2. It also adds three new symptoms, including “distorted sense of blame for self and others”, “persistent negative mood states”, and “engagement in reckless or destructive behaviors” —features often linked to general distress, anhedonia, and dysphoria. Additionally, one symptom was removed, and minor clarifications were made to others.

As for DSM-5, although its revision of PTSD symptom groups was guided by factor analytic research, the inconsistency in findings poses challenges in determining the most concise and effective groupings of symptoms [16]. Therefore, understanding the structural factors is crucial as it directly impacts *diagnostic algorithms* in clinical and research settings, informs knowledge about PTSD’s *comorbidity* with other psychiatric disorders [9,17]. This, in turn, affects disorder *prevalence*, *etiology*, and *intervention* formulation [18,19].

Undoubtedly, PTSD places a considerable medical and economic burden on both individuals and society. Given the importance of efficacious and prompt PTSD treatment, a precise evaluation of PTSD symptoms is of paramount significance. While numerous tools have been formulated for the screening and evaluation of PTSD, the PTSD Checklist (PCL) has surfaced as the predominant self-report instrument employed extensively within both civilian and military cohorts due to its easy administration and because it can be asked secondary to any traumatic event [20].

The original PCL constitutes of 17 self-report items and comprises four distinct versions aligned with the DSM-IV criteria: PCL–Military (PCL-M), PCL–Civilian (PCL-C), and PCL–Specific (PCL-S) [20]. A systematic review of 336 published articles and dissertations employing the PCL assessed the reliability and validity of scores across the three distinct versions, and the findings substantiated those by Weathers et al regarding the PCL’s satisfactory psychometric properties [21].

The original PCL-M, PCL-C, and PCL-S each exhibit three clusters mirroring DSM-IV, while the newly introduced PCL-5 which is a comprehensive single 20-items omnibus measure comprises four clusters mirroring DSM-5: intrusions (Criterion B), avoidance (Criterion C), negative alterations in cognitions and mood (Criterion D), and arousal and reactivity (AAR; Criterion E) [1,20,22,23]. The 20 items include nine that remain the same as in the DSM-IV version of the PCL, five that have undergone minor revisions, three that have been substantially modified, and three new items designed to evaluate the recently introduced PTSD symptoms (*blame, negative emotions, reckless/self-destructive behaviors*). Participants continue to report how much they were bothered by symptoms in the past month; however, the rating scale has been modified from a range of 1–5 to a range of 0–4.

Using PCL-5, an individual meets proxy DSM-5 symptom criteria by considering the items with a score of moderately or above as a symptom endorsed, and then applying the DSM-5 diagnostic criteria of at least one in B (Intrusion), at least one in C (Avoidance), two in D (Negative alterations in cognitions and mood), and two in E (alterations in arousal and reactivity) [22].

Since its release, it is imperative that researchers undertook efforts to perform Exploratory Factor Analysis (EFA) and Confirmatory Factor Analysis (CFA) studies to draw informed and accurate conclusions regarding the PCL-5 instrument’s utility across several languages and settings.

A very recent systematic review looking at factor structural models of PCL-5 summarized the most commonly used models [24]. As shown in S1 Table, the first model is the DSM-5 four-factor Model which includes intrusion, avoidance, negative alterations in cognitions and mood (NACM), and alterations in arousal and reactivity (AAR) similar to the Emotional Numbing (EN) Model used previously in DSM-IV [25]. The second model is the DSM–5 Dysphoria Model which preserves the intrusion and avoidance factors of the EN Model and amalgamates certain AAR and numbing symptoms to formulate the dysphoria factor [26]. The third model is the DSM-5 Dysphoric Arousal Model upholds three factors from the EN Model (intrusion, avoidance and NACM) while segregating the AAR cluster into dysphoric arousal and anxious arousal symptoms [27]. The fourth model is the Anhedonia Model which is similar to the Dysphoric Arousal Model except for the differentiation of the NACM symptoms into negative and positive [28]. The fifth model is the Externalizing Behavior Model which is also similar to the Dysphoric Arousal Model, but it introduces an additional externalizing behaviors factor [29]. The sixth model is the Hybrid Model which amalgamates the factors of all the DSM-5 Models previously mentioned culminating in the identification of seven factors [30].

As evident above, although the PCL-5 Factor Structure Models has been a subject of extensive investigations in diverse studies, nevertheless, there exists a plethora of conflicting outcomes concerning the identification of the optimal model to represent DSM-5 PTSD [12,31].

Supplementary material contains further details on studies backing up specific models, including the Hybrid, Anhedonia, Externalizing, and DSM-5 models, alongside various factor models identified through EFA revealing atypical divergent outcomes.

Methodological variations among studies have been identified as primary contributors to those disparities. These methodological distinctions include variations in population types (e.g., refugees, military personnel, university students, clinical populations, firefighters, healthcare workers and to a lesser extent community samples), sample size fluctuations mostly between 200 and 500, mode of scale administration (e.g., online, face-to-face), language/country, and notably, the nature of the traumas under investigation [24].

### Lebanon

The lifetime prevalence of PTSD in a nationally representative study, published in 2008 in Lebanon, was reported at 3.4% [32]. Subsequent to this study, no nationally representative study has been undertaken to reassess the prevalence, so we undertook an updated estimate of the PTSD prevalence in Lebanon particularly given the heightened risk of PTSD in the Lebanese populace stemming from the compounded impact of the COVID-19 pandemic, the Beirut port blast which was the largest non-nuclear blast recorded in modern history, and the ongoing financial crisis whereby the Lebanese currency lost more than 98 percent of its pre-crisis value with an average inflation of 171.2%—one of the highest rates globally. In this update, 43.5% met the probable DSM-5 PTSD diagnosis using PCL-5. More importantly, a substantial 62.8% of participants screened positive for any disorder (PTSD, Anxiety, or Depression) while 28.10% screened positive for all three disorders.

The observed dearth of research on the behavior of PCL-5 within a general population collectively exposed to multiple major traumas prompted our present study. Our aim is to conduct an Exploratory Factor Analysis of the PCL-5 among a representative sample of the Lebanese population grappling with three major collective traumas. Additionally, the investigation is driven by the recognition that PTSD intricately interacts with depressive and anxiety-related conditions, highlighted by high comorbidity rates.

## Methods

### Procedures

A nationally representative sample of 1,000 Lebanese adults (18+) was obtained according to the Central Administration of Statistics report published by the Ministry of Public Health of Lebanon to reach nationally proportional representation of the age, gender, and governorate. Non-Lebanese individuals and those under the age of 18 were excluded from the study. The phone numbers were obtained from Ipsos’ proprietary database. A random selection from the sample using the Computer Assisted Telephone Interview (CATI) system took place. The interviews were conducted by Ipsos from July to September 2022 following verbal consent. This study was approved by the Institutional Review Board (IRB) committee of the SGHUMC Faculty of Medicine, University of Balamand, Lebanon, which is registered with the U.S Office of Human Research Protections (OHRP) in the Department of Health and Human Services.

### Measures

The instrument used is PCL-5 which is a 20 item instrument that measures the PTSD symptomatology based on the diagnostic criteria outlined in the DSM-5 [22]. The answers were in a form of a 5-point Likert scale to rate each item from 0 (not at all) to 4 (extremely) to indicate the concern about that particular symptom over the past month. A score of 2 (moderately) or greater is considered as an endorsed symptom. The scoring procedures used yielded a dichotomous indicator of diagnostic rank (of probable PTSD).

To ensure clarity and comprehensibility of the items, the PCL-5 was translated into Arabic and back translated to English by the authors. The internal consistency reliability of the PCL-5 in this study was assessed using the Kuder-Richardson 20 (KR-20) coefficient, appropriate for dichotomous items. The KR-20 coefficient was 0.918, indicating high internal consistency reliability for the scale in this study.

### Sample size

Our study utilized a sample size of 1000 participants, which aligns with established guidelines for EFA. For single sample size recommendations, Gorsuch and Kline suggest a minimum of 100 participants, Guilford recommends 200, and Cattell suggests 250, while Comrey and Lee classify 500 as “very good” and 1000 as “excellent” [33–37]. Regarding the N:p ratio, Cattell recommends a ratio of 3:1–6:1, Gorsuch suggests at least 5:1, and Everitt recommends 10:1 [33,36,38]. With 20 variables analyzed in our case, our N:p ratio is 50:1, which exceeds all these standards. Thus, our sample size is robust and ensures reliable and valid factor analysis results.

### Data analysis

Construct validity was examined through Exploratory Factor Analysis (EFA) with an oblique “Promax” rotation [39]. Oblique rotation is suitable for the investigation of latent constructs for which one expects to extract correlated dimensions. We used the Promax with Kaiser Normalization since the correlation between the factors was above 0.6. We performed an EFA as we seek to identify the smallest number of interpretable factors that can adequately explain the correlations among a set of PCL-5 items. According to previous literature, the PCL-5 was designed to assess PTSD-specific symptoms, but overlaps with anxiety and depression are recognized in its structure. To account for this, the EFA was conducted with a theoretical focus on identifying potential distinct dimensions of PTSD symptoms and other related constructs, such as anxiety and depression. This ensures that the factor structure is interpreted in the context of PTSD speficic symptoms while considering the potential contribution of comorbid symptomatology. Kaiser-Meyer-Olkin (KMO) and Bartlett’s test of sphericity were used to test for the appropriateness of factor analysis [40,41]. The KMO measure may vary between 0 and 1; high values of sphericity mean that the variables are correlated and the analysis is feasible. We also generated a scree plot which is used to demonstrate visually the number of significant factors in the scale. The statistical procedures were computed using Stata version 17 and IBM SPSS 25.

## Results

Females constituted approximately half of the participants (51.3%), with a predominant presence in the 18–34-year age range (38%) followed by 26.7% for 35–49, 24.9% for 50–64 and 10.4% for > 65. The majority of individuals were married (60.9%). Employment patterns varied between genders, with a higher percentage of employed men (78.19%) compared to women (36.06%). Geographically, Mount Lebanon had the highest representation at 44.4%, followed by the North (14.2%), the South (9.5%), Beirut (6.3%), and other regions Table 1. This sample proves suitable for validation due to its inherent heterogeneity, encompassing diverse characteristics such as age, education level, marital status, and place of residence.

**Table 1:** Descriptive statistics by genders.

Measurements	Male (487, 48.7%)	Female (513, 51.3%)	Total (1000, 100%)
	N (%)	N (%)	N (%)
**Age**			
*18–34*	130 (26.69%)	250 (48.73%)	380 (38%)
*35–49*	153 (31.42%)	114 (22.22%)	267 (26.7%)
*50–64*	150 (30.8%)	99 (19.3%)	249 (24.9%)
*>65*	54 (11.09%)	50 (9.75%)	104 (10.4%)
**Governorate**			
*Beirut*	27 (5.54%)	36 (7.02%)	63 (6.3%)
*Mount Lebanon*	229 (47.02%)	215 (41.91%)	444 (44.4%)
*North*	59 (12.11%)	83 (16.18%)	142 (14.2%)
*Akkar*	19 (3.90%)	33 (6.43%)	52 (5.2%)
*Bekaa*	45 (9.24%)	27 (5.26%)	72 (7.2%)
*Baalbek-Hermel*	14 (2.87%)	41 (7.99%)	55 (5.5%)
*South*	53 (10.88%)	42 (8.19%)	95 (9.5%)
*Nabatiyeh*	41 (8.42%)	36 (7.02%)	77 (7.7%)
**Marital status**			
*Married*	343 (70.43%)	266 (51.85%)	609 (60.9%)
*Separated, widowed, divorced, never married*	144 (29.57%)	247 (48.15%)	391 (39.1%)
**Employment status**			
*Employed/ Self-Employed*	380 (78.19%)	185 (36.06%)	565 (56.56%)
*Unemployed/ retired/ long-term sick or disabled/ student/ household duties/ volunteer*	106 (21.81%)	328 (63.94%)	434 (43.44%)

For diagnostics, the determinant of the correlation matrix is different than zero, the Bartlett test of sphericity (Chi Square = 7219.886, p-value<0.05) is significant, the Kaiser-Meyer-Olkin measure of sampling adequacy is 0.952 which is above 0.7, meaning that EFA is appropriate for our data.

**Fig 1:**
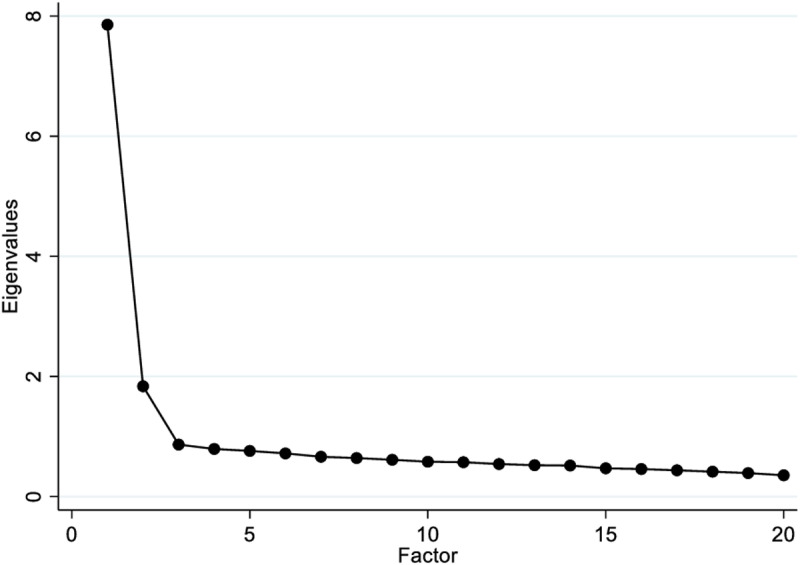
Scree plot.

In order to determine how many factors can explain the analysis, the minimum Eigenvalue for factor retention is above 1 and we were able to retain two factors (7.85611 and 1.83607) which account for 48.461% of the variance. A further examination of the scree plot (Fig 1) suggests a two-factor solution as well where we identified the position which the eigenvalues seem to level off (elbow). The elbow indicates that factors to its left should be retained. In this study, the elbow seems to be positioned after two components which means that only two components are significant. The loading factors of the EFA can be seen in Table 2 and they ranged from 0.546 to 0.757.

**Table 2:** Exploratory factor analysis of PCL-5.

PCL-5 items (in the past 30 days, how much were you bothered by)	Factor 1	Factor 2
Repeated, disturbing, and unwanted memories of the Beirut explosion	**.609**	.392
Repeated, disturbing dreams of the Beirut explosion	**.668**	.421
Feeling or acting as if the Beirut explosion experience was actually happening again	**.681**	.455
Feeling very upset when something reminded you of the Beirut explosion	**.698**	.454
Having strong physical reactions when something reminded you of the Beirut explosion	**.693**	.518
Avoiding memories, thoughts, or feelings related to the Beirut explosion	**.757**	.539
Avoiding external reminders of the Beirut explosion experience	**.718**	.500
Trouble remembering important parts of the Beirut explosion	**.666**	.423
Having strong negative beliefs about yourself, other people, or the world	.420	.593
Blaming yourself or someone else for the Beirut explosion or what happened after it	**.549**	.428
Having strong negative feelings such as fear, horror, anger, guilt, or shame	.555	.657
Loss of interest in activities that you used to enjoy	.480	.608
Feeling distant or cut off from other people	.469	.614
Trouble experiencing positive feelings	.413	.635
Irritable behavior, angry outbursts, or acting aggressively	.407	.644
Taking too many risks or doing things that could cause you harm	.421	.546
Being “superalert” or watchful or on guard	.430	.642
Feeling jumpy or easily startled	.456	.723
Having difficulty concentrating	.418	.652
Trouble falling or staying asleep	.398	.645

## Discussion

To our knowledge, this is the first study that involves the validation of PCL-5 in Lebanon with a representative sample of local population living under multiple stressful conditions.

Our results indicated a two-factor model which support previous literature from DSM-IV and animal models, but not the common reported models in DSM-5. We posit that our first factor yielded items that are specifically answered secondary to the trauma which is the Beirut explosion (9 items), **bolded** in Table 2. The second factor yielded other items which contained questions not trauma specific (11 items). Those 11 items are more likely influenced by the other stressors that the Lebanese are facing including the financial meltdown and the covid-19 pandemic. In our model, the factors are more or less separated into (1) intrusions with avoidance and (2) NACM with alterations in arousal and reactivity with some few exceptions.

In an animal model, Foa found that avoidance serves as a defense mechanism against re-experiencing of traumatic incident, while numbing and desensitization develop as adaptive responses to persistent hyperarousal and stimulation [42]. Similarly, using DSM-IV, Passos and Reichenheim distinguished symptoms into re-experiencing/avoidance and numbing/hyperarousal, indicating a distinction between trauma memory-related reactions (re-experiencing and avoidance) and responses to the threat of trauma (numbing and hyperarousal), while Conybeare supported a slightly different two-dimensional structure [43–45].

It is worth noting that Spitzer et al (2007) had suggested a two-factor model too, which includes consolidating avoidance, numbing, and arousal items into a combined C/D criterion from DSM-IV [46]. This model has been examined twice, with one study favoring its superiority and another study reporting contrasting findings [47,48].

Comorbidity frequently arises in the aftermath of traumatic events, as evidenced by approximately 91% of individuals developing PTSD exhibiting concurrent conditions [49,50]. This aligns with the high comorbidity in our sample as well. The symptomatic overlap between PTSD, depression, and anxiety has been a subject of criticism, contributing to the high comorbidity observed [10,12,46,51–55].

The shared symptoms include anhedonia, irritability, restlessness, a sense of being on edge, difficulty concentrating, heightened startle response, sleep difficulty, impaired trauma recollection, loss of interest, detachment, restricted affect, and sense of foreshortened future disorders [26,52,55–59]. This renders PTSD a heterogeneous disorder, encompassing diverse elements of anxiety and depression hence challenges the construct validity of PTSD due to the considerable similarity [46,60–66].

Some of the PTSD proposed models included dysphoria as a factor offering some perspectives regarding this comorbidity [27,62,67–69]. For example, Simms proposition of the dysphoria was formulated relying on the tripartite model proposed by Clark and Watson to explain the shared characteristics of anxiety and depression [70]. The tripartite model posits three distinct factors: *autonomical arousal* (anxiety characteristic), *anhedonia* (depression characteristic), and a *general distress* factor (anxiety and depression).

Along the same lines, some studies identified associations between somatic depression and dysphoric arousal clusters, while non-somatic depression correlated with NACM and altered arousal and reactivity symptoms [67–69,71,72]. Also, multiple studies found that dysphoria non-specific symptoms exhibit stronger associations with symptoms of MDD compared to more specific PTSD symptoms like intrusions, avoidance and arousal [26,55,56,62,65,73–75].

In addition to the dysphoria factor issue, DSM-5 PTSD have raised concerns about symptom complexity and potential double counting, leading to challenges in diagnosis.

For instance, criterion E1, which involves irritable, angry, or aggressive behavior, represents a behavioral aspect, while criterion D4 pertains to emotional experiences, such as a pervasive negative emotional state, including anger. Distinguishing between these two is likely challenging for patients completing a self-reported symptom checklist which may contribute to an increase in prevalence and comorbidity with depressive disorders due to inflated rates of non-specific symptoms [76,77]. This argument was also supported by Bryant and Fullerton suggesting that elevated symptoms of PTSD after trauma exposure are related to developing symptoms of depression [78,79].

Other researchers found that anhedonia and depressive arousal factors within PTSD demonstrate robust correlations with depression, while avoidance and anxious arousal subscales exhibit strong associations with anxiety measures [80,81].

Similarly, Watson outlined a differentiation between the symptoms D1–D3 and the remaining hyperarousal symptoms of PTSD, namely D4 (hypervigilance) and D5 (exaggerated startle response). The key distinction lies in the nature of D1–D3 symptoms, which encompass general distress or dysphoria, albeit in an agitated or restless manner. On the other hand, D4–D5 symptoms involve anxious arousal typical of fear-based disorders [55].

Various investigations into the dysphoria model, focusing on trauma victims, have aimed to examine the specificity of dysphoria symptoms concerning their associations with trauma exposure, PTSD, and general distress. In this regard, Armour and Shevlin observed that the dysphoria factor exhibited the weakest connection with trauma exposure items [82]. Elklit, after adjusting for depression in three samples, found that dysphoria and other factor loadings were notably diminished, proving that there are nonspecific distress symptoms within PTSD [75].

In light of the challenges surrounding symptom overlap and comorbidity, as well as the issues with factor loading and symptom complexity discussed earlier, our study offers several suggestions for addressing these concerns. These suggestions can be categorized into two main groups: those related to the DSM framework and those that go beyond the DSM.

### DSM-related suggestions

#### One disorder.

As emphasized before, the literature highlights a consideration often overlooked, emphasizing how comorbidity impacts the factor analytic structure of PTSD where it becomes evident that dysphoria symptoms, standing between anxiety and depression as a hybrid diagnosis, may align with the proposed Mixed Anxiety and Depression diagnosis in DSM-5, reflecting less black and white symptom presentations [27,83]. This brings a possible need to reevaluate the diagnostic approach to reduce clinical heterogeneity and comorbidity and invites criticism of the validity of PTSD as a distinct disorder [84].

Demirchyan in her study on earthquake survivors, has proven the significant correlation between PTSD and depression and advocated for the grouping of depression and PTSD into a subclass of “distress disorders” rather than maintaining them as separate disorders [85].

This idea aligns with multiple related work suggesting a common distress factor linking depression, anxiety, and PTSD, challenging the traditional classification of mood and anxiety disorders [55,62,86–88].

#### Concise PTSD diagnosis.

Another suggestion to overcome PTSD heterogeneity is having a concise PTSD diagnosis by removing non-specific dysphoria items which are hypothesized to contribute to the observed high rate of comorbidity between PTSD, anxiety and depression. Multiple researches argues that DSM-5 defines PTSD too broadly while it should be defined by only a few “correct” distinctive features [89–91]. Hence, the advocacy for a narrower and cleaner definition of PTSD, eliminating symptoms overlapping with other disorders and emphasizing a focus on “core elements” is important to make PTSD distinct from anxiety and depression [46,56,73,90,92].

The symptoms identified as having overlapping characteristics encompassed C3-C7 in DSM-IV, along with the anhedonic component designated as the D criteria in DSM-5. These symptoms constituted the second factor in our analysis. We postulated that these symptoms are not directly linked to the primary PTSD resulting from the initial trauma but rather may be associated with anxiety or depression stemming from the Beirut explosion or other concurrent traumas experienced by the Lebanese population, such as those related to COVID-19 and financial challenges.

The effect of this concise diagnosis is also supported by Stein and Spitzer whose epidemiological studies showed that removing dysphoria items for a “narrow-defined diagnosis” significantly reduced PTSD’s diagnostic comorbidity [46,93].

The proposed refinement aligns with the argument that PTSD’s dysphoria symptoms should remain with MDD to maintain a clear distinction and ensure a more accurate representation of the disorder. This approach aims to create a more specific and possibly a clinically meaningful diagnosis, allowing for a targeted focus on the unique aspects of PTSD and preventing potential diagnostic confusion. Not to forget crucially to mention that depression and anxiety are still potential outcomes that can develop independently from and over and above PTSD following traumatic exposure [94,95].

### Non-DSM related suggestions

#### Transdiagnostic treatment approach.

If alternative diagnostic models proposed before (1): *one distress disorder* or (2): *concise definition* are not feasible in future DSM editions, and given the substantial comorbidity, a paradigm shift towards addressing multiple comorbid disorders simultaneously may be necessary for both researchers and clinicians instead of addressing single disorders [96–99]. Transdiagnostic treatments have emerged to target commonalities among emotional disorders, offering a unified protocol for overlapping symptoms [100,101]. Such developments aim to mitigate the challenges posed by diagnostic boundaries and comorbidity, especially among depression, anxiety and PTSD [102].

Whereby, there will be an improved understanding and treatment of reactions to trauma by focusing on individualized presentations and comprehensive assessments considering the assessment of multiple disorders with shared symptom groupings [52,103]. For instance, individuals experiencing a clinically relevant post-trauma reaction may respond best to treatments that address a range of symptoms not only PTSD, but other disorders [103].

For instance, Gros explored transdiagnostic treatments for combat veterans with diverse diagnoses and trauma histories, demonstrating efficacy in reducing both depression and posttraumatic stress [102]. This underscores the need for further research and validation of treatments targeting common symptoms, along with the adoption of tools like the NIMH Research Domain Criteria to enhance comprehension of underlying symptom mechanisms including negative valence systems and positive valence systems [104–106].

#### Assessment challenges.

Scores on self-report scales, such as the PCL-5, suggest a potential overlap between trauma-related symptoms and those associated with depression and anxiety. Some individuals may endorse PTSD symptoms not solely due to the trauma itself but they can be also influenced by emotional distress that could be related to concurrent depression or anxiety.

This challenge is accentuated by the limitation of self-report scales, as highlighted in factor analytic research, where symptoms are not anchored explicitly to the traumatic event as defined by PTSD diagnostic criteria and people experiencing considerable levels of emotional distress might have used this assessment opportunity to express their concerns [107]. This mirrors what we found in our factor analysis where symptoms not specifically asked secondary to the trauma (blast) loaded together and our hypothesis is that the PTSD symptoms are related to other stressors (covid-19 and finance) instead of the blast or simply anxiety or depression.

Therefore, the need for factor analytic studies employing structured diagnostic interviews is emphasized to address this limitation, ensuring a more accurate contextual and temporal linkage of symptoms to trauma exposure and enhancing the precision of PTSD symptom criteria [31]. Additionally, considering alternatives such as face-to-face assessments, rather than relying solely on self-report measures, could provide a more nuanced and accurate understanding of individuals’ experiences, reducing the potential for symptom overlap with depression and anxiety.

### Limitations

The limitations of this study include the fact that it only covers one type of validation, specifically Exploratory Factor Analysis (EFA), which primarily addresses construct validity. As such, the data from this study cannot answer questions related to content validity or criterion validity. Additionally, there are temporal and contextual limitations, as the factor structure may vary in different settings or over time. Furthermore, the use of self-reported PCL-5 data collected via telephone may introduce biases, such as selection bias. Telephone interviews may not be equally accessible to all participants, and those who are willing to participate might differ from the general population.

### Strengths

One of our study’s strengths lie in its use of a representative sample from the general population exposed to various collective traumas, making it one of the largest studies of its kind.

Secondly, our study used EFA which is warranted, specifically when exploring the structure of new PTSD instruments, new symptom criteria, or other unexplored circumstances [108]. The use of EFA is justified as our sample is from a population not previously studied, from a different culture, exposed to multiple stressors and showing high levels of comorbidity. The use of Exploratory Factor Analysis indicates our openness to exploring new aspects of trauma-related outcomes.

Thirdly, our reliance on the well-established PCL-5 instrument adds credibility, given its proven effectiveness in diagnosing PTSD and assessing treatment-related changes.

Finally, numerous factor structures have been proposed for the PCL-5, with Schmitt identifying 15 potential models; however, many of these studies exhibit statistical inaccuracies, such as factor overextraction, leading to a wide range of suggested models with small number of items per factor hence influencing model identification, replication and construct underrepresentation [109]. Our study was reflective of two factor model with a clear distinction between the items loading on the two factors.

### Future suggestion

To advance this research, it is crucial to replicate our study across diverse demographic groups, ensuring the cross-validation of our model in various samples.

While our initial EFA results are promising, it is imperative to replicate them for confirmation by logically conducting a CFA with a sufficiently large sample, as suggested by Gerbing and Hamilton [110]. Moreover, given the high comorbidity between PTSD, depression, and anxiety, it is recommended that future CFA incorporate those other disorders to assess their relationships with specific PTSD factors.

## Conclusion

Ongoing research focuses on developing more intricate models to investigate the role of dysphoria and the structural framework of PTSD. It remains essential for researchers to continue evaluating various models and methods for symptom reduction, striving to balance the intricate nature of PTSD with the need for a concise and practical diagnostic approach. Nevertheless, adding more factors could complicate the diagnosis, resulting in unintended challenges and a potentially more heterogeneous PTSD profile.

As our understanding of the structural nature of PTSD improves and diagnostic measures advance, clinicians and researchers will be better equipped to meet the growing needs of trauma survivors,

especially when dysphoria plays a complex role, particularly in populations exposed to multiple traumas. By refining existing methodologies and incorporating innovative approaches, researchers can make strides in unraveling key aspects of comorbid PTSD and enhancing mental health services.

## Supporting information

S1 Table PCL-5 Factor Structural Models. (DOCX)

S2 File Supplementary Text. (DOCX)

S3 File References. (DOCX)
