# Supplementary material for: Exploratory factor analysis of post traumatic stress disorder checklist for DSM-5: investigating post traumatic stress disorder interconnected dynamics with depression and anxiety in the aftermath of multiple collective stressors
Source: PLoS One. 2025 May 8;20(5):e0323422. doi: 10.1371/journal.pone.0323422 (PMC12061141; doi:10.1371/journal.pone.0323422)
Supplement: S1 Table — (DOCX) [file pone.0323422.s001.docx]

| Criterion & Symptom Number | PTSD Symptom | Model 1: DSM-5 | Model 2: DSM-5 Dysphoria | Model 3: DSM-5 Dysphoric Arousal | Model 4: Anhedonia | Model 5: Externalizing | Model 6: Hybrid |
| --- | --- | --- | --- | --- | --- | --- | --- |
| B1 | Recurrent, involuntary, and intrusive distressing memories | I | I | I | I | I | I |
| B2 | Recurrent distressing dreams | I | I | I | I | I | I |
| B3 | Dissociative reactions (e.g., flashbacks) | I | I | I | I | I | I |
| B4 | Psychological distress to cues | I | I | I | I | I | I |
| B5 | Physiological reactions to cues | I | I | I | I | I | I |
| C1 | Avoidance of distressing memories | A | A | A | A | A | A |
| C2 | Avoidance of external reminders | A | A | A | A | A | A |
| D1 | Inability to remember an important aspect of the traumatic event(s) | NACM | D | NACM | NA | NACM | NA |
| D2 | Negative beliefs | NACM | D | NACM | NA | NACM | NA |
| D3 | Blame himself/herself or others | NACM | D | NACM | NA | NACM | NA |
| D4 | Persistent negative emotional state | NACM | D | NACM | NA | NACM | NA |
| D5 | Markedly diminished interest | NACM | D | NACM | An | NACM | An |
| D6 | Feelings of detachment or estrangement | NACM | D | NACM | An | NACM | An |
| D7 | Persistent inability to experience positive emotions | NACM | D | NACM | An | NACM | An |
| E1 | Irritable behavior and angry outbursts | AAR | D | DA | DA | EB | EB |
| E2 | Reckless or self-destructive behavior | AAR | D | DA | DA | EB | EB |
| E3 | Hypervigilance | AAR | AAR | AA | AA | AA | AA |
| E4 | Exaggerated startle response | AAR | AAR | AA | AA | AA | AA |
| E5 | Problems with concentration | AAR | D | DA | DA | DA | DA |
| E6 | Sleep disturbance | AAR | D | DA | DA | DA | DA |

*I = intrusions; A = avoidance; NACM = negative alterations in cognitions and mood; AAR = alterations in arousal and reactivity; NA = negative affect; An = anhedonia; EB = externalizing behaviors; AA = anxious arousal; D = dysphoria; DA = dysphoric arousal.*
