# Supplementary material for: Exploratory factor analysis of post traumatic stress disorder checklist for DSM-5: investigating post traumatic stress disorder interconnected dynamics with depression and anxiety in the aftermath of multiple collective stressors
Source: PLoS One. 2025 May 8;20(5):e0323422. doi: 10.1371/journal.pone.0323422 (PMC12061141; doi:10.1371/journal.pone.0323422)
Supplement: S2 File — (DOCX) [file pone.0323422.s002.docx]

Although most investigations advocate for the Hybrid model [1-11], others assert the superiority of the Anhedonia model [12], some accepted both Hybrid and Anhedonia [13-15], while some contend in favor of the Externalizing model [16-18], the DSM-5 Model [19-21], and so forth. Furthermore, certain studies have delved into additional Exploratory Factor Analysis, revealing divergent outcomes such as the identification of an Atypical Five Factor Model [22], Four-Factor Model [23-25], three factor model [26], and one factor model [27, 28].
