# Supplementary material for: Exploratory factor analysis of post traumatic stress disorder checklist for DSM-5: investigating post traumatic stress disorder interconnected dynamics with depression and anxiety in the aftermath of multiple collective stressors
Source: PLoS One. 2025 May 8;20(5):e0323422. doi: 10.1371/journal.pone.0323422 (PMC12061141; doi:10.1371/journal.pone.0323422)
Supplement: S3 File — (DOCX) [file pone.0323422.s003.docx]

1. Acquaye HE. Assessing the factor structure of models for posttraumatic stress disorder symptoms in a war-related civilian sample. Counseling outcome research and evaluation. 2018;9(2): 90-101.

2. Armour C, Tsai J, Durham TA, Charak R, Biehn TL, Elhai JD, et al. Dimensional structure of DSM-5 posttraumatic stress symptoms: Support for a hybrid Anhedonia and Externalizing Behaviors model. Journal of psychiatric research. 2015;61: 106-13.

3. Armour C, Contractor A, Shea T, Elhai JD, Pietrzak RH. Factor structure of the PTSD checklist for DSM-5: Relationships among symptom clusters, anger, and impulsivity. The Journal of nervous and mental disease. 2016;204(2): 108-15.

4. Ashbaugh AR, Houle-Johnson S, Herbert C, El-Hage W, Brunet A. Psychometric validation of the English and French versions of the posttraumatic stress disorder checklist for DSM-5 (PCL-5). PloS one. 2016;11(10): e0161645.

5. Caldas SV, Contractor AA, Koh S, Wang L. Factor structure and multi-group measurement invariance of posttraumatic stress disorder symptoms assessed by the PCL-5. Journal of Psychopathology and Behavioral Assessment. 2020;42: 364-76.

6. Carvalho T, da Motta C, Pinto‐Gouveia J. Portuguese version of the Posttraumatic Stress Disorder Checklist for DSM‐5 (PCL‐5): Comparison of latent models and other psychometric analyses. Journal of Clinical Psychology. 2020;76(7): 1267-82.

7. Cheng P, Xu L-Z, Zheng W-H, Ng RM, Zhang L, Li L-J, et al. Psychometric property study of the posttraumatic stress disorder checklist for DSM-5 (PCL-5) in Chinese healthcare workers during the outbreak of corona virus disease 2019. Journal of affective disorders. 2020;277: 368-74.

8. Contractor AA, Caldas SV, Dolan M, Lagdon S, Armour C. PTSD's factor structure and measurement invariance across subgroups with differing count of trauma types. Psychiatry Research. 2018;264: 76-84.

9. Contractor AA, Weiss NH, Dolan M, Mota N. Examination of the structural relations between posttraumatic stress disorder symptoms and reckless/self-destructive behaviors. International Journal of Stress Management. 2020;27(1): 35.

10. Ahmadi A, Galusha JM, Ponder WN, Carbajal J, Schuman DL, Whitworth J, et al. Validation of the PCL-5, PHQ-9, and GAD-7 in a Sample of First Responders. Journal of Occupational and Environmental Medicine. 2023;65(6): 467-76.

11. Grau P, Garnier-Villarreal M, Wetterneck C. An analysis of the latent factor structure of the Posttraumatic Stress Disorder Checklist for DSM-5 (PCL-5) in a PTSD partial hospitalization program. Traumatology. 2019;25(4): 269.

12. Seo W, Cho Y. The Psychometric Properties of the Posttraumatic Stress Disorder Checklist for the DSM‐5 in Korean Adults Exposed to Natural Disasters 1, 2. Japanese Psychological Research. 2023;65(1): 75-84.

13. Blevins CA, Weathers FW, Davis MT, Witte TK, Domino JL. The posttraumatic stress disorder checklist for DSM‐5 (PCL‐5): Development and initial psychometric evaluation. Journal of traumatic stress. 2015;28(6): 489-98.

14. Bovin MJ, Marx BP, Weathers FW, Gallagher MW, Rodriguez P, Schnurr PP, et al. Psychometric properties of the PTSD checklist for diagnostic and statistical manual of mental disorders–fifth edition (PCL-5) in veterans. Psychological assessment. 2016;28(11): 1379.

15. Mordeno IG, Hall BJ. DSM-5-based latent PTSD models: Assessing structural relations with GAD in Filipino post-relocatees. Psychiatry research. 2017;258: 1-8.

16. Boysan M, Guzel Ozdemir P, Ozdemir O, Selvi Y, Yilmaz E, Kaya N. Psychometric properties of the Turkish version of the PTSD Checklist for Diagnostic and Statistical Manual of Mental Disorders, (PCL-5). Psychiatry and Clinical Psychopharmacology. 2017;27(3): 300-10.

17. Tiamiyu MF, Gan Y, Kwiatkowski D, Foreman KC, Dietrich A, Elliott K, et al. Relationships between latent factors of posttraumatic stress disorder and posttraumatic growth. The Journal of Nervous and Mental Disease. 2016;204(5): 344-8.

18. Tsai J, Harpaz-Rotem I, Armour C, Southwick SM, Krystal JH, Pietrzak RH. Dimensional structure of DSM-5 posttraumatic stress disorder symptoms: Results from the National Health and Resilience in Veterans Study. The Journal of clinical psychiatry. 2014;76(5): 4513.

19. Drake-Brooks MM, Hinkson Jr KD, Osteen P, Bryan CJ. Examining the DSM-5 latent structures of posttraumatic stress disorder in a national sample of student veterans. Journal of anxiety disorders. 2020;74: 102262.

20. Durham TA, Byllesby BM, Lv X, Elhai JD, Wang L. Anger as an underlying dimension of posttraumatic stress disorder. Psychiatry Research. 2018;267: 535-40.

21. Biehn TL, Elhai JD, Seligman LD, Tamburrino M, Armour C, Forbes D. Underlying dimensions of DSM-5 posttraumatic stress disorder and major depressive disorder symptoms. Psychological Injury and Law. 2013;6: 290-8.

22. McSweeney LB, Koch EI, Saules KK, Jefferson S. Exploratory factor analysis of Diagnostic and Statistical Manual, criteria for posttraumatic stress disorder. The Journal of Nervous and Mental Disease. 2016;204(1): 9-14.

23. Schmitt TA, Sass DA, Chappelle W, Thompson W. Selecting the “best” factor structure and moving measurement validation forward: An illustration. Journal of personality assessment. 2018;100(4): 345-62.

24. Jiang C, Xue G, Yao S, Zhang X, Chen W, Cheng K, et al. Psychometric properties of the post-traumatic stress disorder checklist for DSM-5 (PCL-5) in Chinese stroke patients. BMC psychiatry. 2023;23(1): 1-12.

25. Orovou E, Theodoropoulou IM, Antoniou E. Psychometric properties of the Post Traumatic Stress Disorder Checklist for DSM-5 (PCL-5) in Greek women after cesarean section. Plos one. 2021;16(8): e0255689.

26. Ferrie O, Richardson T, Smart T, Ellis-Nee C. A validation of the PCL–5 questionnaire for PTSD in primary and secondary care. Psychological Trauma: Theory, Research, Practice, and Policy. 2023;15(5): 853.

27. Kim W-H, Jung Y-E, Roh D, Kim D, Chae J-H, Park JE. Development of Korean Version of PTSD Checklist for DSM-5 (K-PCL-5) and the Short Form (K-PCL-5-S). Psychiatry Investigation. 2022;19(8): 661.

28. Cohen J, Kanuri N, Kieschnick D, Blasey C, Barr Taylor C, Kuhn E, et al., editors. Preliminary evaluation of the psychometric properties of the PTSD checklist for DSM-5. InPoster presented at the 48 th Annual Convention of the Association of Behavior and Cognitive Therapies, Philadelphia, PA, November DOI; 2014.
